# Supplementary figures and images for: TAF7L modulates brown adipose tissue formation
Source: eLife. 2014 May 29;3:e02811. doi: 10.7554/eLife.02811 (PMC4066819; doi:10.7554/eLife.02811)

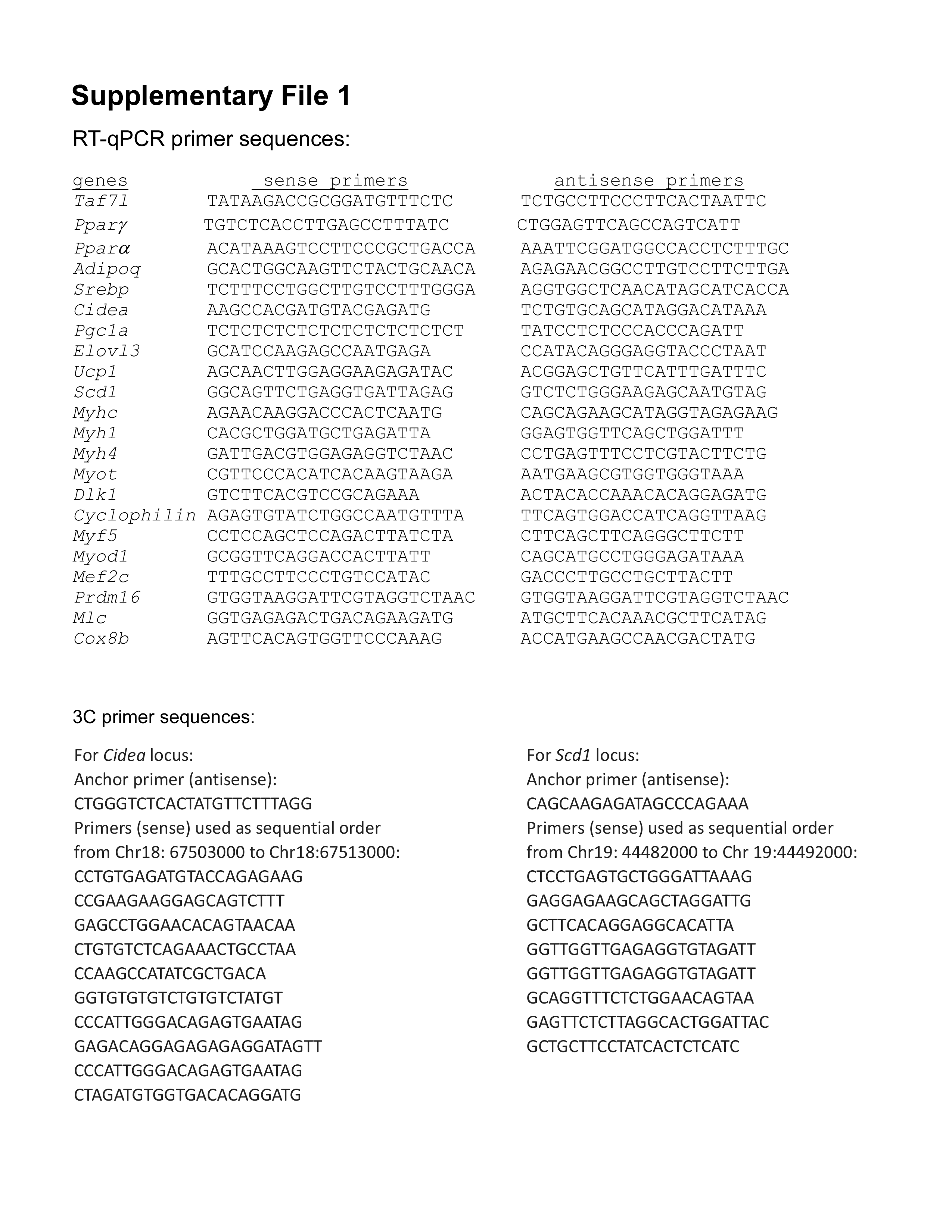

Supplement: Supplementary file 1. — Primer sequences for RT-qPCR experiments (upper panel) and 3C experiments (lower panels). DOI: http://dx.doi.org/10.7554/eLife.02811.011 [file elife02811s001.tif]
